# Chloric Acid

**Published:** 1879-07

**Authors:** D. W. C. Wade

**Affiliations:** Holly, Michigan


### CHLORIC ACID.

By D. W. C. WADE, M.D., Holly, Michigan.

In these days, we may assume that it is conceded that the therapeutical action of easily decomposed salts, administered by way of the stomach, is referable either to the base or the acid, but not to the two as chemically combined, except the amount exceed the decomposing power of the gastric acid while in contact with the salt. I believe it now to be determined that the only acid of the stomach not accidental, is hydrochloric. This acid is capable of decomposing many of the most valuable and frequently prescribed salts. It is known that without decomposition, the action of most of the salts used in medicine would not be in accordance with the object sought to be attained. To illustrate : Carbonates of the alkalies are usually administered on account of the base and not for the carbonic acid. If this acid had as strong affinities as sulphuric acid has, the object would be subverted. Iodide of potassium, bromide of potassium, sulphide of lime, phosphide of zinc, the hypophosphites and many other salts, are not generally given with any reference to the base and had these halogens and acids as strong affinities as has sulphuric acid, the object of their administration would not be accomplished. Again, the action of sulphate of magnesium is like neither the base nor the acid, because it is not decomposed within the system. Neftel says that in fevers the stomach contains no acid, and this assertion is probably true. Such a condition gives rise to many of the symptoms, and may account for many otherwise unexplainable failures in obtaining the effect of remedial measures. Thus, medicines not soluble in water or in alkaline solutions, administered without previous solution during an elevation of temperature, are often worse than wasted. The administration of sulphate of quinia, which is but slightly soluble in water, not mixed with an acid either in or out of the stomach, would but result in disappointment. I wish, therefore, to call the attention of the profession to the frequent desirability of prescribing important therapeutic agents in the form in which we expect the stomach to present them to the circulation, instead of depending upon that capricious organ as a chemical manipulator. Among the salts that are always decomposed in the presence of hydrochloric acid, is chlorate of potassium, and it is probable that the usefulness of this salt as a systemic remedy, and perhaps locally, depends alone upon its acid uncombined. It is to this acid that I invite especial attention. Chloric acid contains one part (equivalent) of chlorine and five parts of oxygen—ClO (O. S.) It is itself easily decomposed, and is a powerful oxidizing agent, and as might be supposed, is an active antiseptic. The uses of chlorate of potassium have been founded partly upon rational and partly upon empirical grounds. I shall not here attempt to discuss the *rationale* of the action of chloric acid, but shall simply in the main advise its use under many circumstances in the place of chlorate of potassium. It is preferable when for any reason we may suppose the stomach is incompetent to prepare the acid from the salt. Chlorate of potassium is only soluble in 16 parts of water, each drachm of its aqueous solution, therefore, containing $3\frac{3}{4}$ grains. The acid can be made more concentrated, and for this reason may be more desirable. The acid is a powerful solvent of the alkaloids and their salts, and has, therefore, convenient combining qualities. Most septicides are acids, and it is probable that the septicidal action of chlorate of potassium resides in its chloric acid and its feeble affinity for the base; hence, the logically greater dependence to be placed upon the acid than its salt in septic affections. It appears to be now a generally accepted opinion that diphtheria is a fermentation disease—at first local and then constitutional. Excellent authorities now claim that, to stay the local action by non-irritating septicides before absorption is possible, invariably ends the affection. It seems likely that the immense reputation of chlorate of potassium in diphtheria has depended upon its power to destroy microscopic life. Chloric acid, in my hands, has proved equally efficient in this disease. The taste of this acid with syrup is much more grateful than when combined with an alkaline base.

Chloric acid may be used to obtain effects from which chlorate of potassium is inert, or comparatively so—as a tonic to aid digestion, a febrifuge in fevers and to overcome the condition called "billiousness." But, equalling all else, the use of chloric acid is in accordance with the exactness of modern methods, and the results are thus so much more likely to be invariable. The time is near at hand when only the unscientific will administer to human beings that which is to be the least depended upon. It should only remain for us to discover how we may avoid error, and there is none greater than to depend upon the stomach as a pharmaceutical laboratory.

I have devised the following formula for the convenient production of chloric acid. The product is not chemically pure, on account of the slight solubility of the by-product bi-tartrate of potassium (cream of tartar), which, according to the United States Dispensatory, is 1 part in 180 of water. This on no account can be urgéd as objectionable. It is to be hoped that the strength of this acid will not be arbitrarily changed by other experimenters, on account of the confusion that results from variable strengths of non-officinal preparations. It would be proper to denominate acids of the strength here given as dilute, in contradiction to concentrated :

I. Chlorate of potassinm .................. 360 grains.
Tartaric acid .......................... 440 grains.
Water ................................ 3 fluid oz.

Or,

II. The salt ............................ 9 troy oz.
The acid ............................ 11 troy oz.
Water ............................... 36 fluid oz.

Or,

III. The salt ........................... 9 avoir oz.
The acid ............................ 11 avoir oz.
Water ............................... 33 fluid oz.

Or,

IV. The salt ........................... 288 grammes.
The acid ........................... 352 grammes.
Water ............................. 1080 c. c.

Dissolve the salt in two-thirds of the water by the aid of heat. Before cooling, add the acid, previously dissolved in one-third of the water. After cooling and completion of the precipitation, decant. Each fluid drachm of the finished product contains the acid of about 15 grains of chlorate of potassium. It is a heavy fluid, and presents the general appearance, taste and smell common to the dilute mineral acids. I have prescribed several gallons in doses for adults of one-fourth to one fluid drachm, largely diluted, apparently without inconvenience.

Take, Dilute chloric acid,
Muriated tincture of iron,
Syrup (flavored).

Quaintities according to age of patients.

Mix, and write : Teaspoonful in water every two hours. (For diphtheria.)

Take, Dilute chloric acid..........2 fluid ounces,
Fluid ext. rhubarb..........1 fluid drachm,
Fluid ext. of gentian........1 fluid ounce,
Syrup to make.............4 fluid ounces.

Mix and write : Teaspoonful in water before meals. (Atonic dyspepsia.)

It is not necessary for me to extend the combination of formulas. A great variety will doubtles be readily suggested to the prescriber.

I apprehend that there is a wide field open for investigation as to the therapeutic application of chloric acid ; among other things, as to its power as an oxidizing agent within the circulation. I do not doubt that the profession will be glad to hear the result of others' experience in its use.

I am not aware that others have produced chloric acid by the formula I have given, have ever prescribed it as a medicine, or that it has ever before appeared as a subject of medical literature.—*Detroit Lancet.*

